# Effects of NIR annealing on the characteristics of al-doped ZnO thin films prepared by RF sputtering

**DOI:** 10.1186/1556-276X-7-294

**Published:** 2012-06-06

**Authors:** Min-Chul Jun, Jung-Hyuk Koh

**Affiliations:** 1 Department of Electronic Materials Engineering, Kwangwoon University, Seoul, 139-701, South Korea

**Keywords:** Al-doped ZnO, Transparent conducting oxide, Thin films, NIR, RF sputtering

## Abstract

Aluminum-doped zinc oxide (AZO) thin films have been deposited on glass substrates by employing radio frequency (RF) sputtering method for transparent conducting oxide applications. For the RF sputtering process, a ZnO:Al_2_O_3_ (2 wt.%) target was employed. In this paper, the effects of near infrared ray (NIR) annealing technique on the structural, optical, and electrical properties of the AZO thin films have been researched. Experimental results showed that NIR annealing affected the microstructure, electrical resistance, and optical transmittance of the AZO thin films. X-ray diffraction analysis revealed that all films have a hexagonal wurtzite crystal structure with the preferentially *c*-axis oriented normal to the substrate surface. Optical transmittance spectra of the AZO thin films exhibited transmittance higher than about 80% within the visible wavelength region, and the optical direct bandgap (*E*_g_) of the AZO films was increased with increasing the NIR energy efficiency.

## Background

Transparent conducting oxide (TCO) has been widely applied for various optoelectronic devices, such as flat-panel and liquid crystal displays [1], organic light-emitting diodes [2], and thin-film solar cells [3]. Most of the TCO materials are based on tin oxide (SnO_2_) and indium tin oxide (In_2_O_3_) with group III elements, such as boron, gallium, indium, or aluminum-doped zinc oxide (AZO) [2]. In particular, Al-doped ZnO-based TCO have been extensively researched because of its low cost and useful properties, such as non-toxicity, excellent electrical and optical properties, and high thermal and chemical stability [4,5].

To fabricate the AZO thin films, several deposition techniques, such as chemical vapor deposition, electron beam evaporation, thermal plasma, pulsed laser deposition, metal organic chemical vapor deposition, sol–gel method, and DC, radio frequency (RF) sputtering have been developed and studied [6-14]. RF sputtering technique is one of the most widely used because of its reproducibility, efficiency, and reliability. There have been lots of reports concerning substrate temperature, oxygen pressure, target to substrate distance, and post-deposition annealing on the AZO thin-film quality using RF sputtering methods [15-18]. Because one of the main factors affecting the deposited film is optical energy, we have employed advanced optical processing by near infrared ray (NIR) annealing method to get better quality AZO thin films. NIR curing method has some advantages. Heat transfer coefficients are high; the process time is short; and the cost of energy is low. Since air is primarily a mixture of oxygen and nitrogen, neither of which absorbs NIR radiation, energy is transferred from the heating source to the sample without heating the surrounding air [19].

In this paper, the NIR annealing effects on the microstructure and the electrical and optical properties of the AZO thin films are reported.

### Experimental details

In this experiment, the AZO thin films were fabricated by RF sputtering on glass substrates from a ZnO:Al_2_O_3_ (98:2) target with 99.999% purity and a 2 in. diameter. The glass substrates (Corning 1737) were ultrasonically cleaned using acetone, methanol, and deionized water, and then dried by blowing nitrogen over them before being introduced into the sputtering chamber. The chamber was initially evacuated to a base pressure under 1 × 10^−6^ Torr, and the deposition was carried out at a working pressure of 2 × 10^−3^ Torr. The argon gas was used as the plasma source, and the gas flow rate was controlled at 40 sccm, using the mass flow controller. The RF power was fixed at 100 W. Prior to the film deposition, pre-sputtering was performed for 10 min to remove any contamination on the target surface. The films were annealed by RTA at 450°C for 10 min and then annealed by a NIR at 20%, 40%, 60%, and 80% energy efficiency for 10 min to investigate the NIR effect.

The thicknesses of the deposited films were investigated by using α-step, and the thickness of the final film was approximately 150 nm. The crystalline structures of the specimens were analyzed by X-ray diffraction (XRD) patterns. XRD 2*θ* scans were carried out by employing an X-ray diffractometer Rigaku with a Cu-*Kα* source (*λ* = 0.154056 nm). The surface microstructure was observed by a scanning electron microscope (SEM). The electrical properties were measured by a four-point probe method, and optical transmittance measurements were carried out using a UV–VIS spectrometer. Photoluminescence (PL) spectra were recorded using a PL spectrometer excited with a 325 nm He-Cd laser at room temperature.

## Discussions

**Figure 1:**
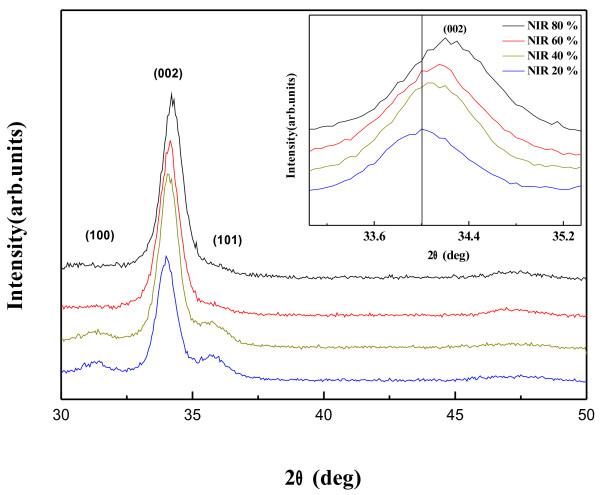
**The XRD scans with Cu*K*α radiation for AZO thin films.**

Figure 1 indicates that the XRD patterns of the AZO thin films are prepared by NIR annealing at 20%, 40%, 60%, and 80% energy efficiency. All films showed strong peaks that confirmed the (002) peaks of hexagonal wurtzite crystal structure, and no metallic Zn or Al characteristic peaks were observed. The relative intensities of the (002) peaks tended to increase with increasing efficiency of NIR, while the (100) and (101) peaks disappeared. The peak intensities of the (002) are displayed in Table 1. It seems that the enhancement of *c*-axis orientation improves the electrical property as a result of the reduction in the scattering of the carriers at the grain boundaries [1]. It is also shown that NIR annealing plays an important role in determining the structure of the AZO thin films.

**Table 1:** Lattice parameter and strain and stress of AZO thin films evaluated from XRD patterns

**Efficiency of NIR (%)**	***I***_**(002)**_	***d***_***hkl***_**(Ǻ)**	***c*(Ǻ)**	**ϵ (%)**	***σ***_**film**_**(GPa)**
20	1,225	2.63455	5.2691	1.3302	−3.0993
40	1,684	2.6308	5.2616	1.1862	−2.7638
60	1,692	2.62335	5.2467	0.8985	−2.0936
80	1,823	2.6196	5.2392	0.7554	−1.7601

The inserted figure shows a magnified 2*θ* region from 33° to 35.4°. It also shows that the (002) diffraction peaks positions of the AZO thin films in the 2*θ* region shift to a higher diffraction angle as the efficiency of NIR increases. The increase of 2*θ* value of the (002) peak may be related to the decrease of lattice parameters that comes from the oxygen defect or the strain caused by crystallization during the NIR process [20,21].

The lattice constant *c* can be calculated by the following formula [22]:

(1) $$d_{hkl}^{2}={\left(\frac{4(h^{2}+k^{2}+hk)}{3a^{2}}+\frac{l^{2}}{c^{2}}\right)}^{-1}$$

where *a* and *c* are the lattice constants, and *d*_*hkl*_ is the crystalline plane distance for indices (*hkl*). According to Equation 1, the lattice constant *c* is equal to 2*d*_*hkl*_ for the (002) diffraction peak. The values of *d*_*hkl*_ and *c* are listed in Table 1 and also are used to confirm the relationship between the crystallinity of the AZO thin films and the efficiency of the NIR process; we investigated the stress in the direction of the *c*-axis. The strain (ϵ) along the *c*-axis in the AZO thin films can be defined as ϵ = [(*c*_film_*– c*_bulk_)/*c*_bulk_, where *c*_bulk_ (5.2 Å) is the unstrained lattice parameter (American Society for Testing and Materials), and *c*_film_ is measured by XRD. Based on the biaxial strain model [23], the stress (*σ*) in the film can be calculated by the following formula, which is valid for a hexagonal lattice:

(2) $$\sigma =\frac{2c^{2}{}_{13}-c_{33}(c_{11}+c_{12})}{2c_{13}}\times \frac{c_{\text{film}}-c_{\text{bulk}}}{c_{\text{bulk}}}$$

The elastic constants *c*_ij_ of single crystalline ZnO are as follow: *c*_11_ = 208.8; *c*_33_ = 213.8; *c*_12_ = 114.7 and *c*_13_ = 104.2 [24]. Equation 2 can be simplified to: *σ*_film_ = -233 × ϵ (GPa); the negative sign means compressive stress. Table 1 shows that the calculated stress and strain for the AZO thin films relaxed with the increasing energy efficiency of the NIR process.

**Figure 2:**
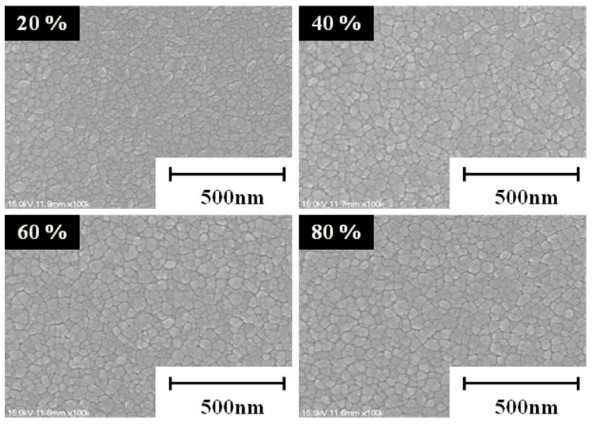
SEM micrographs of AZO thin films post-annealed at different degrees of NIR energy efficiency.

As the microstructure of the AZO thin films can have an influence on the properties of the optical devices, it is very important to investigate the surface morphology of the AZO thin films. Figure 2 shows SEM micrographs of the AZO thin films post-annealed at different degrees of NIR process energy efficiency. It can be seen that the microstructure of all samples became dense, homogeneous, and free of flaws and cracks. By increasing the energy efficiency of the NIR process, the average grain sizes of the AZO thin films increased from 31 to 38 nm, which can be attributed to the improved mobility of the surface adatoms and an increase in cluster formation leading to the coalescence process of small grains from thermal treatment [25]. Moreover, the increased grain size of the AZO thin films due to the increasing annealing temperature has been reported previously [26].

**Figure 3:**
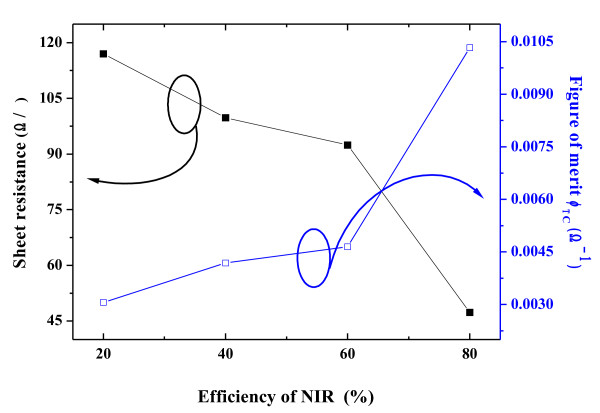
The sheet resistance and figure of merit of AZO thin films.

Figure 3 shows the sheet resistance and figure of merit of the Al-doped ZnO thin films as a function of the energy efficiency of NIR, ranging from 20% to 80%. As shown in Figure 3, the sheet resistance decreased from 117 to 47.3 Ω/□, which can be attributed to an increase in the intrinsic donors and the smooth grain growth process due to a reduction of stress from increasing the energy efficiency of the NIR process. The electrical characteristics of AZO films are related to the number of electrons, the electrons formed from the ionization of the interstitial zinc atom, the extrinsic donor, and the oxygen vacancies. The decrease in sheet resistance of the AZO thin films was mainly affected by the oxygen vacancies in AZO lattice via the NIR annealing process. An oxygen vacancy can offer two electrons, as shown in the following equation [27].

(3) $${\text{O}}_{\text{o}}\to \frac{1}{2}{\text{O}}_{2(\text{g})}{\text{+V}}_{\text{o}}^{\cdot \cdot }\text{+2}{\text{e}}^{\prime }$$

**Figure 4:**
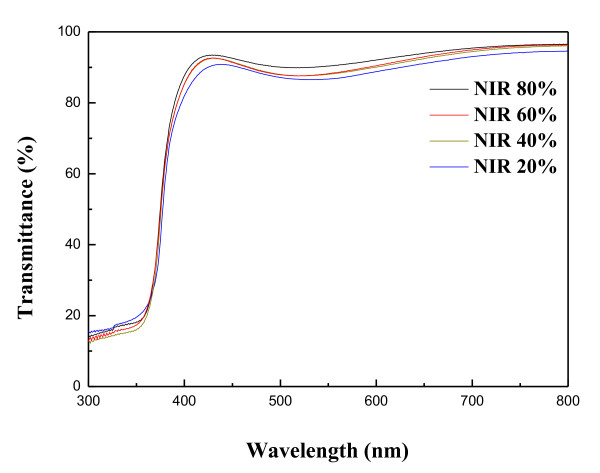
Optical transmittance spectra of AZO thin films.

**Figure 5:**
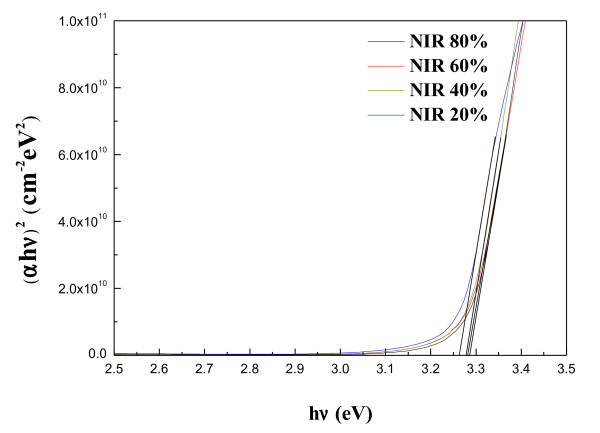
**The plot of (αhν)**^**2**^**versus h*ν*for AZO thin films.**

In general, by increasing the annealing temperature, the resistivity of AZO thin films decreases since the increased grain size produces less grain boundary scattering [1]. The quality of TCO films for their applications in optoelectronic devices can be evaluated by the figure of merit defined by Haacke, which is defined as [28],

(4) $$\phi_{\text{TC}}=\frac{T^{10}}{R_{s}}$$

where *T* is the average optical transmittance in the visible region (400 to 800 nm), and *R*_*s*_ is the sheet resistance of the AZO thin films. The higher values of the figure of merit represent the better performance of the TCO films. Figure 3 shows that the figure of merit increases from 3.06 × 10^–3^ Ω^–1^ to 1.03 × 10^–2^ Ω^–1^ with the increase of the NIR energy efficiency. The film quality estimated by figure of merits by Haacke method showed that the NIR method can improve AZO-film quality, and this value can be comparable to the result already reported in ITO film [29]. Optical transmittance spectra of the AZO thin films post-annealed by NIR at various energy efficiencies of 20% to 80% in the wavelength from 300 to 800 nm were compared in Figure 4. All of the films exhibited a higher transmittance than 80% in the visible region. As the wavelength of incident light decreased to the ultraviolet region, the transmittance of the AZO thin films decreased with a sharp fundamental absorption edge at around 380 nm of the wavelength. From Figure 4, it was also found that the absorption edges slightly shift toward the lower wavelength value as a result of increasing the energy efficiency of the NIR process. This shift is confirmed by representing the absorbance squared versus h*ν* in Figure 5. The absorption coefficient data were used to determine the optical bandgap, *E*_g_, using the relation [30]:

(5) $$\alpha \text{h}v\approx {\left(\text{h}v-E_{\text{g}}\right)}^{1/2}$$

where h*ν* is the photon energy. The absorption coefficient *α* was obtained from the transmittance data by employing the relation *α* = (1/*d*) ln(1/*T*), where *d* and *T* are the thickness and the transmittance of the films, respectively. Accordingly, the optical bandgap *E*_g_ can be obtained by extrapolating downwards the corresponding straight lines to the photon energy axis in the Tauc plot [31]. The optical bandgap of the AZO thin films was increased in accord with an increase of NIR energy efficiency from 3.26 to 3.29 eV. NIR treatment increased the optical bandgap of the AZO thin films. Typically, the blue shift of the absorption edge of the AZO thin films is associated with an increase of the carrier concentration blocking the lowest states in the conduction band, which is well known as the Burstein-Moss effect [32,33].

**Figure 6:**
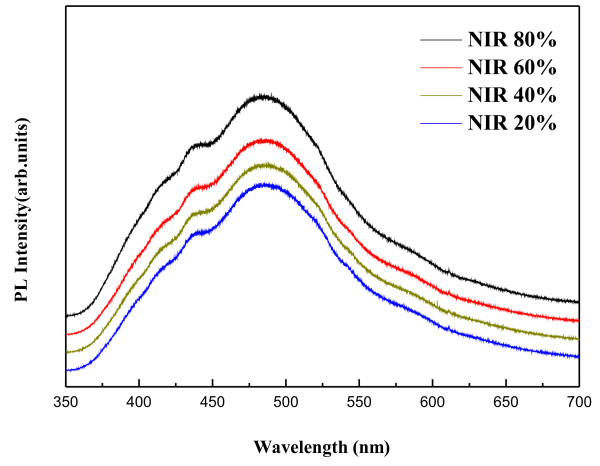
PL spectra of AZO thin films.

Figure 6 indicates the room-temperature PL spectra of the Al-doped ZnO thin films post-annealed at various degrees of NIR energy efficiency. In general, the luminescence property of the AZO thin film is closely related to the stoichiometry and crystal quality of AZO. All AZO thin films show the broad green emission peaks. The intensity of green emission is found to increase as the NIR energy efficiency is increased since the green emission depends on the relative concentrations of free electrons and defects created by oxygen vacancies in its lattice as a result of heating treatment [34]. This result is consistent with the XRD analysis, and it indicates that the crystal quality of the AZO thin films can be controlled by adjusting the energy efficiency of the NIR process.

## Conclusions

In this paper, the RF-sputtered AZO thin films deposited on glass substrates and post-annealed by the NIR process at the energy efficiency levels of 20%, 40%, 60%, and 80% have been discussed. The effects of the NIR process on the optical, structural, and electrical properties of the AZO thin films have been studied. The films were found to be *c-*axis oriented. The AZO thin film with the optimal crystal quality had the lowest sheet resistance at 47.3 Ω/□. We obtained a high transmittance in the visible range, and the optical bandgap energy *E*_opt_ obtained was *E*_opt_ = 3.26 to 3.29 eV for the films put through the NIR process.

In conclusion, it was observed that the structural, morphological, electrical, and optical characteristics of AZO thin films can be improved by the NIR process, and that the NIR process seems to be an effective annealing method for the TCO process.

## Authors’ contributions

MCJ carried out the experiments and measurements. MCJ and JHK performed the analysis and prepared the manuscript. JHK had been guiding the research. All authors read and approved the final manuscript.
